# Arabidopsis CROWDED NUCLEI (CRWN) proteins are required for nuclear size control and heterochromatin organization

**DOI:** 10.1186/1471-2229-13-200

**Published:** 2013-12-05

**Authors:** Haiyi Wang, Travis A Dittmer, Eric J Richards

**Affiliations:** 1 Boyce Thompson Institute for Plant Research, 533 Tower Road, Ithaca, NY 14853, USA; 2 National Cancer Institute, National Institutes of Health, Bethesda, MD 20892, USA

**Keywords:** Nuclear organization, Nuclear morphology, Nuclear DNA density, Heterochromatin

## Abstract

**Background:**

Plant nuclei superficially resemble animal and fungal nuclei, but the machinery and processes that underlie nuclear organization in these eukaryotic lineages appear to be evolutionarily distinct. Among the candidates for nuclear architectural elements in plants are coiled-coil proteins in the NMCP (Nuclear Matrix Constituent Protein) family. Using genetic and cytological approaches, we dissect the function of the four NMCP family proteins in Arabidopsis encoded by the *CRWN* genes, which were originally named *LINC* (*LITTLE NUCLEI*).

**Results:**

CRWN proteins are essential for viability as evidenced by the inability to recover mutants that have disruptions in all four *CRWN* genes. Mutants deficient in different combinations of the four CRWN paralogs exhibit altered nuclear organization, including reduced nuclear size, aberrant nuclear shape and abnormal spatial organization of constitutive heterochromatin. Our results demonstrate functional diversification among CRWN paralogs; CRWN1 plays the predominant role in control of nuclear size and shape followed by CRWN4. Proper chromocenter organization is most sensitive to the deficiency of CRWN4. The reduction in nuclear volume in *crwn* mutants in the absence of a commensurate reduction in endoreduplication levels leads to an increase in average nuclear DNA density.

**Conclusions:**

Our findings indicate that CRWN proteins are important architectural components of plant nuclei that play diverse roles in both heterochromatin organization and the control of nuclear morphology.

## Background

The cellular components and processes that specify nuclear size, shape and internal organization are poorly understood, particularly in flowering plants. Despite similarities at the gross morphological level among all eukaryotic nuclei, such as a double-membrane boundary perforated with nuclear pores, most of the proteins known to affect nuclear structure in animals are not evolutionarily conserved and are consequently difficult to recognize or absent entirely in plant proteomes [1-3]. These observations indicate that the machinery, and perhaps the principles, specifying nuclear organization in flowering plants are distinct from those operating in animals and represent a convergent evolutionary path to a canonical nuclear organization in eukaryotic cells [4].

We demonstrated previously [5,6] that two paralogous Arabidopsis coiled-coil proteins, originally named LITTLE NUCLEI 1 and 2 (LINC1 & 2), play important roles in specifying nuclear shape and size. Supporting this conclusion, Sakamoto and Takagi recently reported that disruption of *LINC4*, another of the four paralogous genes in this family, leads to reduced nuclear size and loss of elongated nuclear shape in differentiated cells, mirroring the phenotype of *linc1* mutants [7]. These proteins are closely related to NMCP1, Nuclear Matrix Constituent Protein 1, originally identified as a protein residing on the periphery of carrot nuclei and a component of the salt-resistant nuclear matrix [8]. Although NMCP1 and related proteins are plant-specific and share no significant amino acid similarity to lamins, their tripartite structure with an extensive central coiled-coil domain and their localization at the nuclear periphery suggest that NMCP1-related plant proteins might be functional analogs of this core component of the animal nuclear lamina [9,10]. More recent computational analysis [11], however, has suggested that the NMCP class of plant proteins shares more structural similarities to myosins or paramyosins than to lamins.

Here, we extend our reverse genetic analysis to encompass all four members of the Arabidopsis NMCP-related protein family, which we have renamed CRWN (CROWDED NUCLEI) to avoid confusion with the acronym LINC (LINKER of NUCLEOSKELETON and CYTOSKELETON) that refers to SUN-KASH protein linkages that bridge the inner and outer nuclear membrane [12-16]. Our findings demonstrate that CRWN proteins are essential for viability, and our analyses uncover complex functional diversification among CRWN proteins with regards to their effects on whole-plant morphology, nuclear size, and the spatial organization of constitutive heterochromatin aggregates (chromocenters) in interphase nuclei. We found that CRWN1 plays the most prominent role among CRWN paralogs in controlling nuclear size, while CRWN4 has the most important role in controlling the distribution and number of heterochromatic chromocenters. The reduced nuclear size in *crwn* mutants is not matched by a commensurate reduction in endopolyploid levels, resulting in increased nuclear DNA densities (mass per unit volume) up to four-fold higher than wild type levels.

## Results

We performed a phylogenetic analysis of Arabidopsis CRWN proteins and their homologues in other species to begin our investigation of the potential diversification within this family. The predicted Arabidopsis proteome contains four closely related CRWN proteins (CRWN1 through 4) that share 30-40% amino acid identity; no other Arabidopsis proteins with extended regions of significant amino acid identity to CRWN proteins were found. Similar proteins were found in other plant species, but interestingly, no fungi or animal CRWN homologues were identified from searches of protein databases. In addition, CRWN homologues were absent in the predicted proteome of the green algae Chlamydomonas and Volvox.

**Figure 1:**
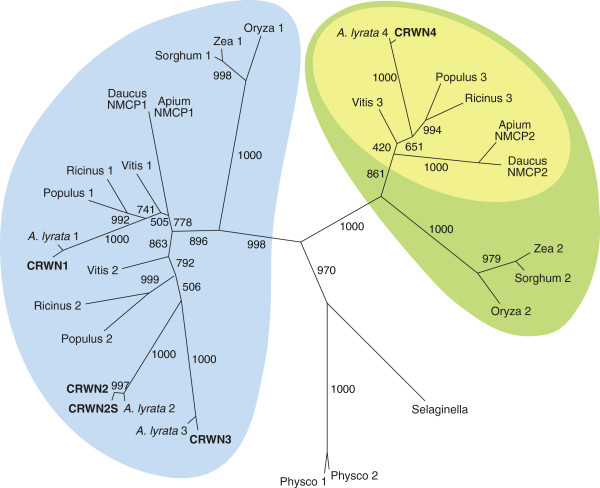
**Phylogenetic relationships among CRWN proteins.** A maximum likelihood tree of CRWN homologs constructed from an alignment of amino acid sequences that correspond to the coiled-coil domains. Bootstrap values (of 1000 replicates) are indicated on each branch. The *A. thaliana* CRWN proteins are indicated in bold, and homologs are labeled with the genus name and an assigned number (Additional file 1: Table S1). Two major clades are marked by blue and green; the yellow inset oval indicates a subgroup of CRWN4-like proteins from dicots that lack the conserved C-terminal domain (Additional file 2).

We constructed a phylogram of CRWN proteins and related plant homologs using a maximum likelihood algorithm (Figure 1 and Additional file 1: Table S1). The tree features two major clades distinct from CRWN homologues in two basal plants, *Selaginella moellendorffii* and *Physcomitrella patens*. One clade includes three of the Arabidopsis paralogs, CRWN1, CRWN2 and CRWN3, while CRWN4 belongs to the other clade. Within each clade, the monocot proteins, represented by maize, sorghum and rice, group independently from the dicot proteins. Only two CRWN paralogs exist in these monocots – one CRWN1-like and one CRWN4-like. However, certain dicot species, such as Arabidopsis, poplar, grape, and castor bean, contain multiple copies of CRWN1-like proteins. The dicot CRWN4-like proteins are also distinct from their monocot counterparts in lacking a conserved amino acid motif at the extreme C-terminus (yellow inset in Figure 1 and Additional file 2).

### Genetic redundancy in the CRWN family

The inference that CRWN4 and related proteins are divergent from members of the CRWN1-containing clade was supported by genetic analyses to dissect the functions of the *CRWN* paralogs. We used Agrobacterium T-DNA insertion alleles to study the effects of inactivating different combinations of *CRWN* genes [17]. Previously, we demonstrated that the *crwn1-1* and *crwn2-1* T-DNA alleles severely reduce or eliminate transcription downstream of the T-DNA insertion [5]. Here, we performed transcript analysis by RT-PCR for the *crwn3-1* and *crwn4-1* alleles used in this study (Additional file 3). For *crwn3-1*, some transcript was detected downstream of the insertion; however, no transcript could be detected using primers that flanked the insertion. The *crwn4-1* insertion blocked transcription downstream of the T-DNA. The lack of full-length *CRWN* transcripts from homozygous mutant lines indicates that all four mutations used in this study are likely to be loss-of-function alleles. We note that the *CRWN* genes have similar developmental gene expression patterns: the steady-state abundance of transcripts for all four paralogs peak in proliferating tissues (http://bar.utoronto.ca/efp/cgi-bin/efpWeb.cgi) [18].

**Figure 2:**
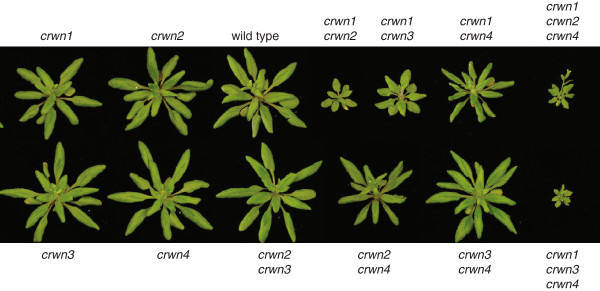
**Whole plant phenotypes of *crwn *mutants.** Leaf rosette structure of one month-old plants imaged just after initiation of flowering. Representative plants for the various genotypes are compared to a wild type (WT) Columbia plant. All plants were grown in parallel and photographed at the same magnification; the diameter of a WT rosette at flowering is ca. 8 cm.

Mutant plants carrying single insertions were intercrossed and progeny carrying homozygous insertions in different combinations were recovered. Figure 2 shows the whole-plant phenotype of the viable mutants at the rosette stage just after the transition to flowering. Plants carrying a mutation in any single *CRWN* gene had phenotypes similar to wild-type Columbia plants, as did the double *crwn2 crwn3* and *crwn3 crwn4* mutants. The *crwn2 crwn4* and *crwn1 crwn4* double mutants exhibited slightly smaller rosettes, while the remaining double mutants, *crwn1 crwn2* and *crwn1 crwn3*, displayed markedly smaller rosette sizes. We were able to recover only two of the four triple mutants - *crwn1 crwn2 crwn4* and *crwn1 crwn3 crwn4*, both of which were extremely stunted and set few seed.

Our inability to isolate a mutant combining alleles in all four *CRWN* genes indicates that at least one functional CRWN protein is required for viability. Triple mutant plants carrying only CRWN2 or CRWN3 were extremely stunted, but still viable. This result suggests that CRWN2 or CRWN3 alone can cover the minimum requirements for the entire CRWN protein family. However, plants carrying only *CRWN1* or only *CRWN4* were not recovered, suggesting that CRWN1 and CRWN4 are specialized and that neither protein alone can express the full range of functions of the CRWN protein family.

### CRWN proteins are required to maintain proper nuclear size and shape

**Figure 3:**
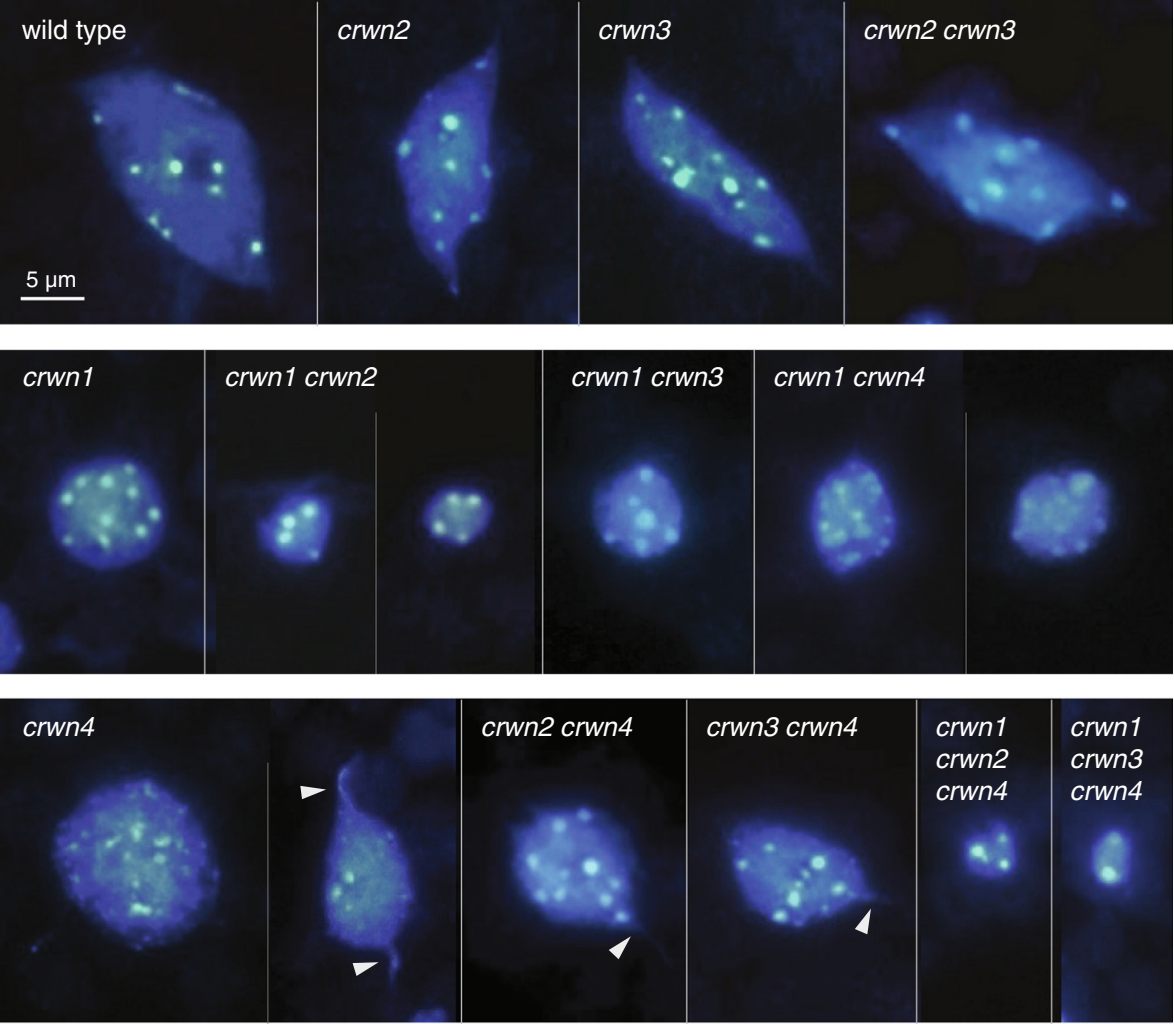
**Nuclear phenotypes of *crwn *mutants.** Images of representative DAPI-stained adult leaf cell nuclei from wild type and the twelve viable *crwn* mutants. The top row contains the wild type control and *crwn2*, *crwn3* and *crwn2 crwn3* mutants with normal nuclear morphology phenotypes. The middle row shows nuclear phenotypes of *crwn1* and double mutants containing a *crwn1* mutation. The bottom row displays nuclear phenotypes from *crwn4* mutants, as well as higher-order mutants containing a *crwn4* mutation. The arrowheads highlight thin projections from *crwn4* nuclei. A 5 μm size bar is shown in the upper left inset; all images are shown at the same magnification.

**Figure 4:**
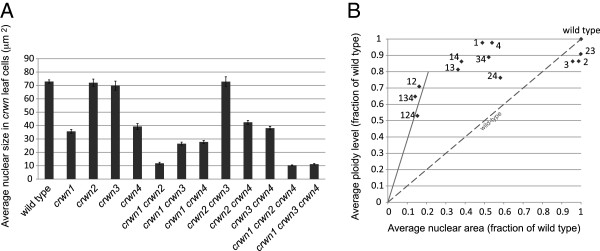
**The effects of *crwn *mutations on nuclear size and nuclear DNA density in leaf cells. (A)** Developmentally matched rosette leaves from approximately one month-old plants were fixed, cells isolated and stained with DAPI, and nuclei imaged using epifluorescence microscopy. The average areas of randomly-selected individual nuclei (n = 32–108) were determined for each genotype. Error bars indicated standard error of the mean. **(B)** Nuclear area measurements from panel **A** (see also, Additional file 4) were converted to relative values and were plotted against average endopolyploidy level for each genotype expressed as a fraction of the wild-type value. The average endopolyploidy levels (Additional file 4) were measured by flow cytometry using corresponding leaf samples. The diagonal dashed line indicates the expected linear relationship between nuclear area and endopolyploid level observed in wild-type plants. Note that panel **A** measures nuclear area but the isolated nuclei are flattened under a coverslip to a uniform thickness (see Additional file 6) and therefore nuclear area is proportional to and therefore a proxy for nuclear volume. The numbers next to the symbols indicate the corresponding *crwn* genotype. Data points above the dashed line indicate an elevated nuclear DNA density relative to wild type. The solid line corresponds to a nuclear DNA density four-times that of wild type.

We next observed *crwn* mutant nuclei from adult leaf tissue to determine the role of different CRWN proteins in specifying nuclear size and shape. Among the single mutants, a deficiency of *CRWN1* or *CRWN4* reduced nuclear size (Figures 3 and 4A; Additional file 4), while loss of CRWN2 or CRWN3 had no effect. Combining a *crwn1* mutation with a *crwn2* or *crwn3* mutation had a synergistic effect on nuclear size, suggesting that CRWN1 function overlaps, at least partially, with those of CRWN2 and CRWN3. Double mutant combinations containing *crwn4* and either *crwn2* or *crwn3* did not show additive phenotypes but rather resembled *crwn4*. In contrast, combination of a *crwn1* with a *crwn4* mutation had an additive effect on nuclear size. These findings indicate that CRWN1 and CRWN4 are the major determinants of nuclear size among the CRWN paralogs. Further, the additive effects of *crwn1* and *crwn4* mutations suggest CRWN4 acts independently from CRWN1, consistent with their distinct phylogenetic grouping (Figure 1) and the genetic analysis shown in Figure 2.

CRWN proteins are required for development or maintenance of the elongated spindle shapes which characterize larger nuclei in differentiated wild-type cells [19]. We previously reported that a deficiency of *CRWN1* causes nuclei in all cells to adopt the spherical shape characteristic of proliferating tissue at root and shoot apices [5]. The present study confirmed the importance of CRWN1 for nuclear shape differentiation and also uncovered a similar role for CRWN4 (Figure 3), a conclusion also reached recently by Sakamoto and Takagi [7]. Nuclei from *crwn4* leaf tissue often have irregular margins and are more spherically shaped, compared to wild-type nuclei (Additional file 5). However, *crwn4* nuclei are less uniformly round in comparison to *crwn1* nuclei, particularly larger *crwn4* nuclei. Further, *crwn4* nuclei occasionally contain thin projections that appear to be drawn from the nuclear surface (arrowheads in Figure 3).

### Loss of CRWN proteins affects nuclear DNA packing density

The direct correlation between endopolyploidy and nuclear size in wild-type Arabidopsis cells [20] prompted us to examine this relationship within the *crwn* mutants. We measured the average endopolyploidy level of nuclei from the same adult leaves harvested for the nuclear size analysis shown in Figure 4A (see also Additional file 4). Some *crwn* mutants showed a decrease in endopolyploid levels, particularly the *crwn* triple mutants and the *crwn1 crwn2* double mutant, but the remaining *crwn* genotypes had average endopolyploidy levels that approached wild-type levels (Figure 4B). The dashed line in Figure 4B depicts the expected nuclear size change in response to a reduction in endopolyploidy based on the established one-to-one relationship between nuclear volume (approximated by nuclear area in our measurements of isolated and flattened leaf cell nuclei, Additional file 6) and DNA content in wild type plants. With the exception of *crwn2* and *crwn3*, the *crwn* mutations caused a more pronounced reduction in nuclear size than predicted from the observed endopolyploidy level. As a consequence, *crwn* mutants display a spectrum of nuclear DNA densities, ranging from wild-type values in *crwn2* and *crwn3* mutants to four-fold higher densities in *crwn1 crwn2* double mutants and the two viable *crwn* triple mutants.

**Figure 5:**
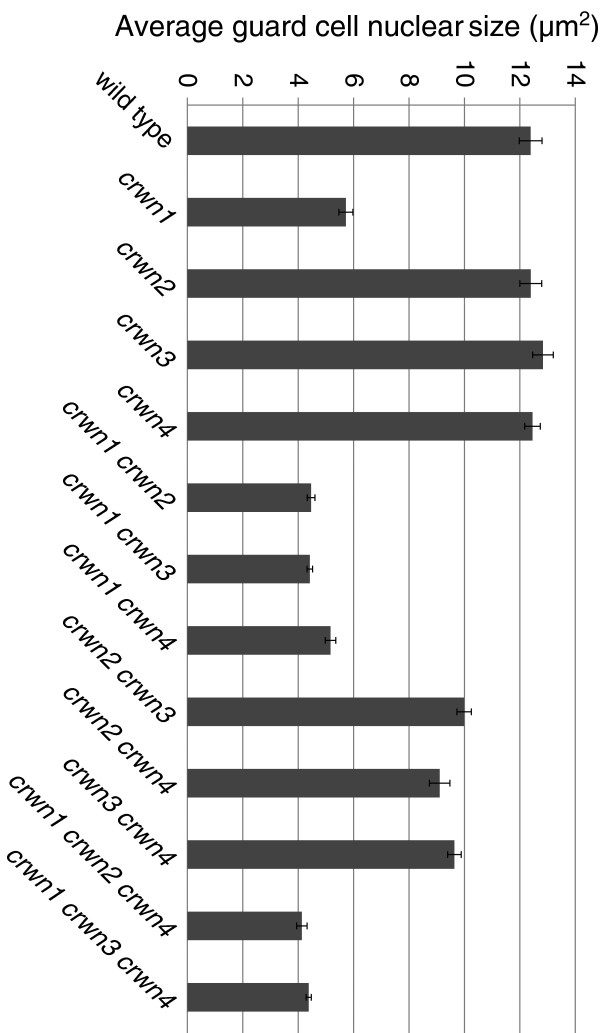
**Average leaf guard cell nuclear sizes in *crwn *mutants.** Two week-old plants were harvested, fixed, stained with DAPI, and leaf guard cell nuclei were imaged using confocal laser scanning microscopy. The area of randomly-selected individual nuclei (n = 19–29) were determined for each genotype. Error bars indicated standard error of the mean.

We then investigated the relationship between nuclear size and DNA content by examining the effects of different *crwn* genotypes on nuclear size in leaf guard cells, a diploid cell type where endopolyploidy is not a factor [21]. *crwn1* mutant guard cell nuclei were smaller than nuclei in wild type cells with an area approximately one-half of the wild type value, corresponding to a volume difference of approximately threefold assuming a roughly spherical shape to nuclei in the cell (Figure 5). Double and triple mutants lacking *CRWN1* displayed nuclear sizes similar to the *crwn1* single mutant. Consistent with their effects on nuclear size shown in Figure 4A, neither the *crwn2* nor *crwn3* mutation affected nuclear size in guard cells. Interestingly, the size of nuclei in *crwn4* guard cells was also unaffected, in contrast to the effect seen in a population of adult leaf cells (Figure 4A). However, *crwn2 crwn3*, *crwn2 crwn4*, and *crwn3 crwn4* double mutants had nuclei approximately 20% smaller than those seen in wild-type guard cells, suggesting some functional redundancy among CRWN2, CRWN3 and CRWN4 proteins. Overall, our results indicate that *CRWN1* plays the major role in affecting nuclear size in the absence of changes in endopolyploidy.

### CRWN4 maintains interphase chromocenter integrity and organization

**Figure 6:**
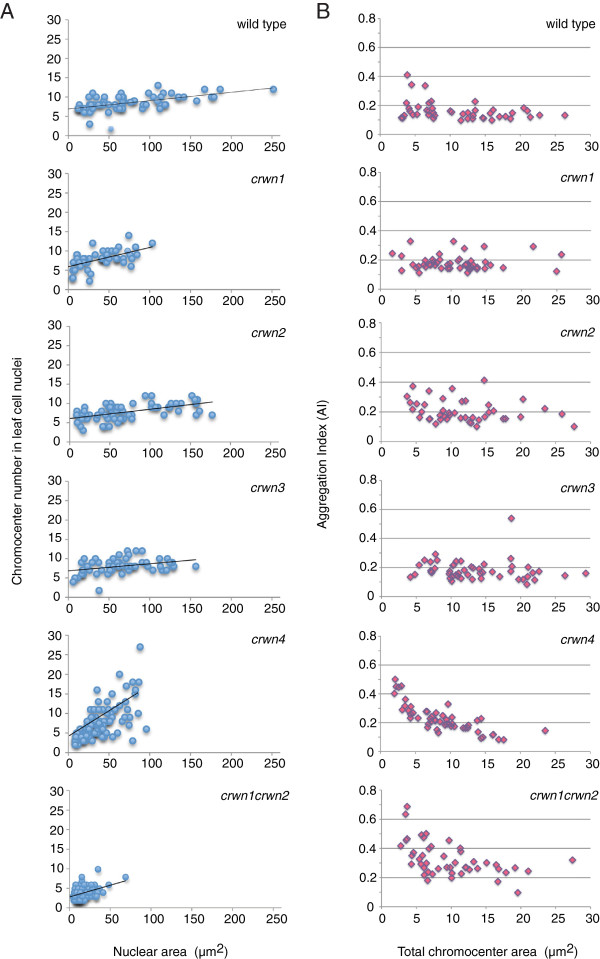
**Chromocenter morphology changes in *crwn *mutants. (A)** Nuclei were imaged from developmentally matched rosette leaves from approximately one month-old plants, cells isolated and stained with DAPI, and nuclei imaged using epifluorescence microscopy. The area and chromocenter number of randomly-selected individual nuclei (n = 47–132) were determined for each genotype, and chromocenter number was plotted against nuclear area. A linear regression line showing the relationship between chromocenter number and nuclear size (as a proxy for endopolyploidy level) was plotted for each genotype. **(B)** Nuclei were imaged fully expanded rosette leaves from approximately one month-old plants and the chromocenter Aggregation Index (AI) was plotted against the total chromocenter area (μm^2^).

Considering the dramatic effects of *crwn* mutations on nuclear size and morphology, we turned our attention to the role of CRWN proteins on the internal organization of the nucleus. A conspicuous feature of Arabidopsis interphase nuclei are discrete foci of heterochromatin, or chromocenters, visualized as bright spots after staining with fluorescent DNA-intercalating dyes [22]. A typical interphase nucleus contains approximately ten chromocenters corresponding to the number of diploid chromosomes (2n = 10) [23]. Chromocenter number remains fairly constant over a wide range of nuclear sizes and endopolyploid levels (2n to 16n), most likely as a result of lateral association of sister chromatids after endoreduplication [24,25]. We found that the average chromocenter number in *crwn1*, *crwn2* and *crwn3* leaf cell nuclei was similar to that seen in wild-type leaf cell nuclei (Figure 6A) and did not change dramatically as a function of nuclear size. In *crwn4* nuclei, however, chromocenter number was strongly correlated with nuclear size (Figure 6A): smaller nuclei contained fewer chromocenters than the wild-type value of ~9, while larger, presumably endopolyploid, *crwn4* nuclei exhibited a wide range of chromocenter numbers (2–27). A similar pattern was observed in double mutants containing the *crwn4* mutation (Additional file 7). In contrast, double mutants containing the *crwn1* allele paired with another *crwn* mutation displayed a reduced average chromocenter number with a weaker association with nuclear size (Figure 6A and Additional file 7).

To explore the chromocenter phenotype in more detail, we developed a statistic, referred to as an aggregation index (AI) (see Methods), to characterize the distribution of visible DAPI-bright spots within interphase nuclei. The value of this index ranges from 0 to 1, reflecting both the number of distinct chromocenter spots and the uniformity of their size distribution. The expected AI for wild-type nuclei containing 10 equally sized chromocenters is 0.1, while clustering of chromocenters into fewer but larger aggregates will lead to a higher AI value. A dispersal of chromocenters into smaller heterochromatic satellites will push the AI lower. For a given chromocenter number, a skewed CC size distribution is associated with a larger AI compared to when each CC is equally sized. As shown in Figure 6B, the AI index of wild-type nuclei averaged close to 0.1 and was not affected significantly by nuclear size. The absence of a significant correlation between AI and nuclear size indicates that chromocenter organization remains constant across different endopolyploidy levels in wild-type nuclei. A similar pattern was observed for the *crwn1*, *crwn2*, and *crwn3* mutant samples. In contrast, combining *crwn1* and *crwn2* mutations led to an approximately two-fold higher AI over a range of nuclear sizes, consistent with the two-fold reduction in chromocenter number via aggregation in *crwn1 crwn2* mutants. A different pattern was displayed in the *crwn4* sample, which displayed a negative correlation between AI and nuclear size. This result suggests a tendency for chromocenters to aggregate in smaller *crwn4* nuclei and to become dispersed in larger *crwn4* nuclei. The reduction in chromocenter number in *crwn1 crwn2* and *crwn4* mutants with smaller nuclei could reflect the aggregation of individual chromocenters in the limited confines of these nuclei, but a similar clustering does not occur in small wild-type nuclei, arguing that small nuclear dimensions alone are insufficient to cause clustering. The variability in chromocenter size and number in *crwn* mutant nuclei suggests that CRWN proteins are required for proper organization of heterochromatin in interphase nuclei.

**Figure 7:**
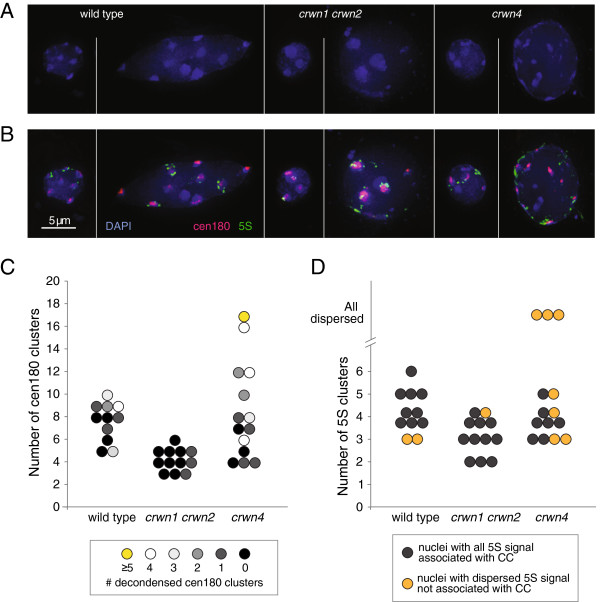
**Chromocenter organization is altered in *crwn1 crwn2 *and *crwn4 *mutants. (A and B)** Fluorescence *in situ* hybridization of representative small and large nuclei from wild type, *crwn1 crwn2* and *crwn4* cells prepared from leaves of adult plants after bolting. Blue: DAPI, pink: 180-bp centromere repeats, green: 5S RNA genes, bar, 5 μm. **(C)** The distribution of nuclei based on the number of centromere repeat clusters in different genotypes. Each circle represents an individual nucleus in the FISH experiment. The color indicates the level of decondensation of the centromere signal. **(D)** The distribution of nuclei based on the number of 5S RNA repeat clusters in different genotypes. Each circle represents an individual nucleus. The color indicates the level of dispersion of the 5S repeat signal.

We tested this hypothesis by visualizing the spatial arrangement of chromocenter-associated genomic regions in *crwn1 crwn2* and *crwn4* mutants. Arabidopsis chromocenters are comprised of large segments of repetitive DNA such as the tandemly-arrayed centromeric and 5S RNA repeats located within pericentromeric regions [23]. Using fluorescent *in situ* hybridization (FISH), we examined the spatial organization of the major 180-bp centromeric tandem repeat and the 5S RNA gene arrays in both large and small nuclei from wild-type, *crwn1 crwn2* and *crwn4* plants (Figure 7A, B). The centromeric and 5S RNA repeats were co-localized with the DAPI-bright spots in both small and large wild-type nuclei, confirming previous reports that these sequences are normally compartmentalized within chromocenters at the nuclear periphery [26] (see Additional file 8: Movie S1). It was common to find a decondensed centromere signal at several chromocenters in wild-type nuclei; however, decondensed centromeric repeat clusters were infrequently observed in *crwn1 crwn2* nuclei and the total number of clusters was reduced (Figure 7C) (also see Additional file 8: Movie S2). These findings indicate that there is a compaction of the centromere repeat arrays within coalesced chromocenters in *crwn1 crwn2* nuclei. In contrast, the number of discrete centromere repeat clusters visible in *crwn4* nuclei was more variable, and decondensed signals were often seen in nuclei with numerous clusters. This pattern is consistent with the hypothesis that chromocenters become dispersed in larger *crwn4* nuclei. A similar but more pronounced trend was seen for the 5S RNA gene arrays (Figure 7B, D), which were dispersed outside chromocenter aggregates in roughly one-half of the *crwn4* nuclei. We note that the dispersed 5S RNA gene signal remained localized to the nuclear periphery (see Additional file 8: Movie S3). The apparent dispersal of chromocenters in larger *crwn4* nuclei and the mis-positioning of centromeric and 5S RNA repeats outside of the chromocenter indicates that higher-order organization of heterochromatin breaks down in interphase in the absence of CRWN4.

## Discussion

Our results demonstrate that the CRWN family is essential for viability in Arabidopsis and required for proper nuclear organization. Redundancy and diversification exists among the four CRWN paralogs, which belong to a plant-specific family of nuclear coiled-coil proteins that forms two clades: one including CRWN1, CRWN2 and CRWN3 and the other containing CRWN4 (Figure 1). The divergence of CRWN4 relative to the other CRWN paralogs, also reported by Kimura et al. (2010) [10] and Ciska et al. (2013) [9], is supported by our genetic analysis. First, the inviability of triple mutants containing either *CRWN1* or *CRWN4* alone indicates that CRWN1 and CRWN4 possess non-overlapping functions (Figure 2). Second, loss-of-function mutations in *CRWN1* and *CRWN4* have distinct phenotypes; for example, *crwn1* is the only *crwn* mutation that affects nuclear size in diploid guard cells (Figure 5), while the dispersed chromocenter phenotype is unique to the *crwn4* mutant (Figures 3 and 7). Finally, the functional distinction between the two CRWN clades is supported by taxa, such as rice and maize, which contain only two CRWN-like proteins: one CRWN1-like and one CRWN4-like.

The phenotypic effects of combining mutations in different *CRWN* genes demonstrates that all four paralogs are involved in specifying nuclear size in adult cells. A deficiency in CRWN1 led to a dramatic reduction in nuclear size independent of an effect on endopolyploidy (Figure 4). Loss of CRWN4 also reduced nuclear size in leaf cells (Figure 4) as was recently shown by Sakamoto and Takagi (2013) [7]. Interestingly, *crwn4* nuclei in leaf guard cells were not reduced in size (Figure 5), despite the fact that *CRWN4* is expressed in this cell type (http://bar.utoronto.ca/efp/cgi-bin/efpWeb.cgi) [18], suggesting cell-type specific requirements exist for different CRWN proteins. Combining a *crwn1* loss-of-function mutation with a deficiency in any of the remaining three paralogs causes a further reduction in nuclear size in leaf cells. The additive effect on nuclear size indicates that CRWN2 and CRWN3 share overlapping functions with CRWN1, although loss of CRWN2 and/or CRWN3 has no effect on nuclear size or endopolyploidy in leaf cells when a functional *CRWN1* allele is present. We previously reported that the *crwn2-1* allele caused a reduction in nuclear size in leaf cells [5]; the reason for the different behavior displayed in this study is unknown, but we have noted variability in nuclear phenotypes among different *crwn2* mutant lines. Regardless, it is clear that CRWN1 plays the major role in adult leaf tissue among the three paralogs in the CRWN1-like clade. This situation might reflect the higher level of expression of *CRWN1* in leaf tissue compared to *CRWN2* and *CRWN3* – ca. 30 FPKM (fragment per kilobase/million mapped RNA-seq reads) for *CRWN1*, with 4× and 2× less expression from *CRWN2* and *CRWN3*, respectively (data not shown). Alternatively, the different contributions of *CRWN1*-like genes could also result from structural differences among the encoded proteins.

Our data indicate that the primary phenotype of *crwn* mutants is a reduction in nuclear size and that the reduction in endopolyploidy observed in double and triple *crwn* mutants is a secondary effect in response to reduced nuclear size. First, mutation of either *CRWN1* or *CRWN4* had an effect on nuclear size in leaf cells without affecting endopolyploidy (Figure 4) (see also [7]). Second, effects on endopolyploid levels were only seen in mutants that contained two or more *crwn* mutations and exhibited severely reduced nuclear size. These considerations indicate that loss of CRWN activity alters the relationship between DNA content and nuclear volume, leading to a higher than normal nuclear DNA density. In the most severely affected mutants, the nuclear DNA density reaches four times the level seen in wild-type cells. It is intriguing that the *crwn* mutants with the most abnormal whole-plant dwarfing phenotypes, including *crwn1 crwn2* and the viable triple *crwn* mutants, are the ones with the highest average nuclear DNA density.

We hypothesize that the CRWN proteins are required for nuclear expansion after nuclear reformation in telophase [27-29], and that the loss of these proteins, especially in combination, results in an elevated nuclear DNA density. Evidence supporting this mechanism comes from a recent report demonstrating that *crwn1 crwn2* cotyledon nuclei expand more slowly than their wild type counterparts during the first 72 hours of seed germination, a period normally characterized by a ca. 10-fold expansion of nuclear size in the absence of endoreduplication [30]. Interestingly, the rate of contraction of *crwn1 crwn2* embryonic cotyledon nuclei during seed maturation is also reduced relative to wild type. These observations suggest that CRWN proteins are involved in remodeling nuclear structure during interphase in response to developmental and environmental cues. Similarly, a constraint in nuclear expansion associated with endoreduplication [20,31] could explain the limit on endopolyploid levels observed in high-order *crwn* mutants.

The large coiled-coil domains that comprise the central region of all four CRWN proteins point toward a structural role in the nucleus. Recent analysis based on secondary structure for analogues of Arabidopsis coiled-coil proteins indicates that CRWN proteins are candidates for paramyosin homologues [11]. Paramyosin is a structural protein found in invertebrate muscles, where it forms the core of thick filaments and bundles with myosin motors. Deficiency of paramyosin, encoded by the *unc-15* gene in *C. elegans*, leads to shortened, mis-formed and apparently fragile thick filaments in the nematode’s muscles [32]. The structural similarity to paramyosin suggests models for CRWN action as architectural components of the nucleoskeleton. Models of this type predict that *crwn* mutant nuclei would have a less sound structural foundation and be more prone to breakage or distortion by intracellular forces (e.g., exerted by the cytoskeleton or at programmed nuclear expansion/contraction transitions), as seen in animal cells when lamin is disrupted [33] or down-regulated [34]. The irregular margins and thin projections seen among *crwn4* nuclei are consistent with this structural integrity model. These predictions, however, are not consistent with the smaller, round nuclei in *crwn1* mutants that do not adopt the elongated spindle shapes typical of wild-type nuclei in many cell types. These considerations suggest alternative models wherein the CRWN1 protein might establish flexible hinge regions or expansion zones in the nucleoskeleton, facilitating nuclear size changes that accompany endopolyploidy and other developmental transitions. We note that both nucleoporin 136 [35,36] and LINC complex mutants [15] in Arabidopsis also lead to more spherical nuclear shapes, suggesting that CRWN proteins might interact with these complexes at the nuclear periphery.

Deficiency of CRWN proteins also affects chromocenter organization (Figures 6 and 7). We previously reported that chromocenter number decreases approximately two-fold in *crwn1 crwn2* nuclei [5], and we confirmed these results here while extending our analysis to the entire *CRWN* gene family. One unexpected finding was the wide variation in chromocenter number in *crwn4* nuclei and the direct correlation between chromocenter number and nuclear size. The reduction in chromocenter number in *crwn1 crwn2* mutants, as well as *crwn4* mutants with smaller nuclei, is consistent with aggregation of individual chromocenters. The *in situ* hybridization data shown in Figure 7 further support this conclusion. Further, we demonstrated that chromocenter organization was disrupted in larger *crwn4* nuclei as evidenced by the dispersed signals seen for the 5S RNA genes, and to a less extent, the centromere repeat arrays. Considering that chromocenters are localized primarily to the nuclear periphery [23,26] (see Additional file 8: Movies S1-3) where CRWN proteins are also located [5-7], CRWN proteins might play a direct role in ensuring proper heterochromatin organization. In such models, CRWN1 and CRWN2 would act to prevent chromocenter aggregation and CRWN4 would exert a complementary effect to maintain chromocenter integrity.

The four distinct phenotypes displayed by *crwn* mutants – reduced nuclear size, altered nuclear shape, elevated nuclear DNA density and abnormal organization of constitutive heterochromatin – highlight a functional connection among these different aspects of nuclear architecture. The lack of whole-plant phenotypes of Arabidopsis *crwn1* and *crwn4* mutants is remarkable in light of the dramatic nuclear changes occurring in these mutants and underscores plants’ plasticity in their ability to execute an apparently normal developmental program in spite of these nuclear changes. The diversity of CRWN proteins and the ability to work with viable *crwn* mutants that exhibit dramatic nuclear phenotypes will facilitate the elucidation of the mechanisms through which these essential proteins exert their effects on nuclear organization.

## Conclusions

This study addresses fundamental questions about how plant cells specify and control the morphology of their nuclei and its relationship with internal chromatin organization. We conducted a comprehensive reverse genetics study of the *CRWN* gene family in Arabidopsis, which encode NMCP-class proteins implicated in nuclear morphology and organization. We demonstrated that CRWN proteins are essential for viability, and in the process, uncovered a surprisingly high degree of functional diversity among the CRWN proteins. CRWN1 and CRWN4 are the major determinants of nuclear size and shape, and we hypothesize that deficiency in CRWN proteins leads to defects in nuclear expansion and remodeling. One consequence of this deficiency is an increase in nuclear DNA density as endoreduplication levels are not affected except in the most extreme cases (e.g., *crwn1 crwn2* and the viable triple mutants). Our findings also demonstrated that CRWN4 plays a role in maintenance of heterochromatin organization in interphase nuclei. The specificity of the nuclear morphological and higher-order chromatin organization defects seen in *crwn* mutants reveals the interplay between nuclear morphology and the three-dimensional packaging of the genome.

## Methods

### Plant materials and growth conditions

All T-DNA insertion alleles used in this study are from the SALK collection [17] in strain Columbia, and single mutant lines were originally obtained from the Arabidopsis Biological Resource Center (ABRC) at The Ohio State University. Plants were grown in long-day lighting conditions (16 h of light/8 h of dark) at 23°C on soil (Metro-Mix 360, SunGro, Vancouver) in environmental growth chambers. Genotyping of individual T-DNA alleles was performed by standard PCR using the following pairs of allele-specific primers: SALK_025347 (*crwn1-1*), 5′-TGC CTT CTC CTC GCT TTT CAA-3′ and 5′-TGC GTG AAT GGG AAA GAA AGT TG-3′; SALK_076653 (*crwn2-1*), 5′-GAA GCT CAT TGC TAG AGA AGG GG-3′ and 5′-AAC GCT GAT CGT TCA TGT TCC A-3′; SALK_099283 (*crwn3-1*), 5′-TTC TGC ATC TTG ACA CCA TCC AA-3′ and 5′-TCG TCG ACT AGT TAA CAA AAT CA-3′; SALK_079296 (*crwn4-1*), 5′-CGC AAA GCC TTC GAA GAC AAA-3′ and 5′-GCT TCA GCC AGC ATT TCA AGC-3′.

### Phylogenetic tree construction

Amino acids similar to CRWN1 were downloaded from public databases (see Additional file 1: Table S1). The program ClustalX was used to align the amino acid sequences. The tree in Figure 1 is based on an alignment of the region of highest conservation across all amino acid sequences, corresponding to the coiled-coil domains (amino acids 64 to 651 in CRWN1). A maximum likelihood tree was constructed using Phylip 3.69 with 1000 bootstrap replicates.

### Diploid guard cell nuclear area measurement

Two-week old seedlings were harvested and fixed in 3:1 acetic acid:ethanol. Nuclei in the fixed tissue were stained using DAPI (10 μg/ml, 2 minutes), and guard cell nuclei were imaged using laser scanning confocal microscopy (Leica SP5). Images were taken at the focal plane with the maximum nuclear area, and the resulting images were processed using ImageJ software.

### Leaf nuclei isolation and imaging

Nuclei were isolated from the fifth true leaf of adult plants harvested after initiation of flowering stem elongation (stem height ≥ vegetative rosette diameter). Therefore, the tissues were developmentally matched across the different genotypes. Mesophyll cells predominated but other cell types were present. Each harvested leaf was bisected and one half used for the nuclear area measurement, while the remaining half leaf was processed for flow cytometry measurements (see below). Leaf tissue was fixed using a 3:1 acetic acid:ethanol solution and tissues were rehydrated in 100 mM sodium citrate buffer pH 4.8 for 15 min followed by incubation in digestion buffer (0.03% cytohelicase, 0.03% pectolyase, and 0.03% cellulase Onozuka RS in 100 mM sodium citrate buffer pH 4.8) for 2 hours at 37°C. Digested tissue was carefully homogenized by pipetting, centrifuged briefly at low speed, and resuspended with 100 mM sodium citrate buffer pH 4.8; this cycle was repeated three times and the final pellet was resuspended in 3:1 acetic acid:ethanol. The resulting suspension of nuclei were pipetted onto microscope slides, dried for ca. 1 min, and stained with 10 μg/ml 4′,6-diamidino-2-phenylindole (DAPI). A Leica DM 5500 epifluorescence microscope was used to image the nuclei, and the nuclear area was measured from digital images using ImageJ software after manual tracing of nuclear boundaries. Note that the chromocenter number versus leaf cell nuclear area scatter plots shown in Figure 6A were generated in a separate experiment from the one shown in Figure 4, but developmentally matched leaf tissues were harvested as described above.

### Aggregation index measurement

Tissue from adult leaves was harvested and prepared as described for FISH (see below). Rather than performing the *in situ* hybridization step, nuclei were stained with DAPI and imaged using optical sectioning microcopy. Projections of the processed images were analyzed using ImageJ software to identify chromocenters. Briefly, the images were manually manipulated when necessary to adjust the local threshold and chromocenter area and number were assigned using the Analyze Particle function of the software. The aggregation index (AI) was calculated using the following equation: AI = Σ (S_i_/S_total_)^2^; where i = 1, …, n; S_i_ = the area of chromocenter i; S_total_ = the total area of all chromocenters in the nucleus.

### Flow cytometry

Bisected tissue from the fifth true leaf of adult plants were harvested (see above) and immersed in magnesium sulfate buffer [37] and chopped with razor blades in a petri dish. The resulting suspension was filtered through a nylon mesh (diameter = 30 μm; Partec Cell Trics®, Münster, Germany). The nuclear suspension was incubated with RNAse A (Ribonuclease A, from bovine pancreas, Sigma, St. Louis, MO, USA) (50 μg/ml) on ice for 15 min and stained with propidium iodide (50 μl/ml) in the dark for 6 hours. Average ploidy level for each genotype was calculated based on the peaks generated from an analytical flow cytometer (Accuri 6 model, Accuri cytometers, Ann Arbor, MI, USA).

### Fluorescent *in situ* hybridization (FISH)

Fully expanded adult leaf tissue was harvested less than a week after flowering stem elongation, and fixed in Buffer A [38] with 4% formaldehyde at room temperature with agitation for >1 hour. After rinsing with Buffer A, the tissue was chopped repeatedly with razor blades until a homogenous texture was achieved. A clear nuclear suspension was pipetted from the leaf debris and used for fluorescence *in situ* hybridization as described by Golubovskaya et al. (2002) [38]. The centromere probe 5′- Cy5-GGTTGCGGTTTAAGTTCTTATACTCAATC -3′ was synthesized by Integrated DNA Technologies (Coralsville, IA, USA), and the 5S probe was amplified from genomic DNA using primers 5′-CTNCCNGGNAGNTCACCC-3′ and 5′-CCTNGTGNTGNANCCCTC-3′, followed by labeling using a nick translation protocol and Rhodamine labeled dCTP (Roche; Indianapolis, IN, USA).

## Competing interests

The authors declare that they have no competing interests.

## Authors’ contributions

HW planned and performed the experiments, prepared the figures, and contributed to the preparation of the manuscript. TAD provided technical advice, generated genetic reagents and contributed to the preparation of the manuscript. EJR supervised the project and wrote the manuscript. All authors read and approved the final manuscript.

## Supplementary Material

Additional file 1: Table S1 CRWN-like proteins used in this study. The first column shows the abbreviated name of the protein used in alignment to construct the tree shown in Figure 1. The remaining columns indicate the identity and the source of each protein sequence. Click here for file

Additional file 2 **Amino acid sequences comprising the extreme C-termini of 28 CRWN-like proteins, including ten CRWN4-like proteins.** The similarity in this region, which falls outside of the coiled-coil domains, reinforces the topology of the tree shown in Figure 1. All of the proteins within the CRWN1-like clade, as well as the Physcomitrella homologs, contain a conserved C-terminal motif and a group of acidic residues approximately 25 amino acids from the end of the protein. Monocot CRWN4-like proteins contain a region with similar features but these conserved motifs are absent in CRWN4-like proteins from dicots (denoted by the yellow oval in Figure 1). Click here for file

Additional file 3 **Transcript analysis of the *crwn3-1 *** **and *crwn4-1 *** **alleles used in this study.** Reverse transcription-PCR results investigating the effect of T-DNA insertions on the transcription of *CRWN3* and *CRWN4*. Panel **A** shows that a *CRWN3* transcript is produced from the wild-type allele but not from the *crwn3-1* allele using primers spanning the T-DNA insertion site. Panel **B** demonstrates that some transcription can be detected downstream of the insertion site from the *crwn3-1* allele using RT-PCR and a primer set recognizing sequences 3’ of the insertion site. Panel **C** indicates that the T-DNA insertion in the *crwn4-1* allele blocks transcription. Amplification of cDNA from cyclophilin and *Actin2* were used as positive controls. M, marker lanes; + RT (plus reverse transcriptase); - RT (no reverse transcriptase). Information on the oligonucleotide primers used in these experiments is shown at the bottom of the figure. Our previous data [5] indicated that the *crwn1-1* and *crwn2-1* alleles block transcription downstream of the T-DNA insertion site in the sixth exon of both genes. Click here for file

Additional file 4: Table S2 The nuclear phenotype data for *crwn* mutants used to construct Figure 4 is displayed in tabular form. The average endopolyploid level (ave. ploidy level) was determined by flow cytometry as described in Methods. The actual measurements were converted to relative measurements (fraction of wt, third column) using the wild type (wt) values for normalization. The average nuclear size (ave. nuclear size ± standard error of the mean) corresponds to data from Figure 4A. The fifth column normalizes these size values to the wild type values. Click here for file

Additional file 5 **Nuclear shape changes in *crwn1 *** **and *crwn4 *** **mutants.** Images of representative DAPI-stained adult leaf cell nuclei from wild type, *crwn1* and *crwn4* mutants were processed by ImageJ software to determine the circularity index (4π · Area/(perimeter)^2^), as well as a shape index (perimeter/π · major axis)^2^. Nuclei that deviate from a perfect circle (1.0) show a lower circularity index. The shape index highlights different types of deviations from the round shape. Nuclei in the *crwn1* sample show a shape index close to 1.0, indicating consistently round nuclei. The reduced (relative to 1.0) shape indices in the wild-type sample across all nuclear sizes indicate uniformly elongated nuclear shapes. The elevated shape indices characteristic of larger *crwn4* nuclei result from the presence of thin projections from the surface of otherwise round nuclei. Click here for file

Additional file 6 **Leaf nuclear preparation and confocal imaging reveals a consistent nuclear thickness across a range of nuclear sizes.** Mature leaves were harvested from five individuals in a F2 population segregating both *crwn1* and *crwn2* mutations (F2 plants of a *crwn1 crwn2* x wild type cross). Consequently, the sample captured a range of nuclear shapes and sizes. The nuclei were fixed, isolated, and prepared for imaging as described for Figure 4. Following DAPI staining, the three-dimensional signal of different nuclei were recorded and reconstructed using a Leica SP5 confocal microscope. The area of each nucleus was measured using ImageJ, while the thickness of each nucleus was determined by the number and thickness of steps on the z-axis necessary to move from the top to the bottom of each nucleus. The different colored dots on the graph correspond to different slides imaged in this experiment. The results indicate that our preparation and imaging procedure generates nuclei with a relatively uniform thickness, mostly in the 2–3 micrometer range, regardless of the size and shape of the nuclei. Further, this thickness is consistent across individual slides. Click here for file

Additional file 7 **Chromocenter changes in *crwn *** **double mutants.** Nuclei were harvested from developmentally matched rosette leaves from approximately one month-old plants, stained with DAPI, and imaged using epifluorescence microscopy. The area and chromocenter number of randomly-selected individual nuclei (n = 41–52) were determined for each genotype, and chromocenter number was plotted against nuclear area. A linear regression line showing the relationship between chromocenter number and nuclear size (as a proxy for endopolyploidy level) was plotted for each genotype. Click here for file

Additional file 8: Movies S1-3 Three-dimensional reconstruction of nuclei imaged in the fluorescence *in situ* hybridization experiment. Representative nuclei from wild type **(Movie S1)**, *crwn1 crwn2***(Movie S2)** and *crwn4***(Movie S3)** leaf cells were processed by FISH as described in Figure 7. Blue: DAPI, pink: 180-bp centromere repeats, green: 5S RNA genes. The 180-bp centromere repeat and the 5S RNA gene signals were localized to the nuclear periphery in all genotypes. Click here for file
