# Supplementary material for: Arabidopsis CROWDED NUCLEI (CRWN) proteins are required for nuclear size control and heterochromatin organization
Source: BMC Plant Biol. 2013 Dec 5;13:200. doi: 10.1186/1471-2229-13-200 (PMC3922879; doi:10.1186/1471-2229-13-200)
Supplement: Additional file 1: Table S1 — CRWN-like proteins used in this study. The first column shows the abbreviated name of the protein used in alignment to construct the tree shown in Figure 1. The remaining columns indicate the identity and the source of each protein sequence. [file 1471-2229-13-200-S1.pdf]

Table S1

Protein	Species name	Locus name or Sequence ID	Database source*
Physco 1	*Physcomitrella patens*	Pp1s200_64V6.1	JGI
Physco 2		Pp1s76_81V6.1	
Selaginella	*Selaginella moellendorffii*	XP_002993584.1	GenBank
Apium NMCP1	*Apium graveolens*	BAF64421.1	GenBank
Apium NMCP2		BAI67716.1	
Daucus NMCP1	*Daucus carota*	BAA20407.1	
Daucus NMCP2		BAI67718.1	
Ricinus 1	*Ricinus communis*	XP_002525969.1	GenBank
Ricinus 2		XP_002524388.1	
Ricinus 3		XP_002530596.1	
Vitis 1	*Vitis vinifera*	CAO49297.1 (GSVIVG01031076001)	GenBank & (JGI)
Vitis 2		CAN74873.1 (GSVIVT01011972001)	
Vitis 3		CAO17747.1 (GSVIVT01007428001)	
Populus 1	*Populus trichocarpa*	XP_002329317.1 (Potri.017G111400.2)	GenBank & (JGI)
Populus 2		XP_002312375.1 (Potri.008G114800.1)	
Populus 3		XP_002317738.1 (Potri.012G034300.1)	
CRWN1	*Arabidopsis thaliana*	At1g67230.1	TAIR
CRWN2		At1g13220.2	
CRWN2S		At1g13220.1 alternative splicing variant	
CRWN3		At1g68790.1	
CRWN4		At5g65770.1	
A_lyrata 1	*Arabidopsis lyrata*	scaffold_2:12,299,939..12,304,385	JGI
A_lyrata 2		471477	JGI
A_lyrata 3		476006	JGI
A_lyrata 4		scaffold_803322.1.1	Ensembl Genomes
Zea 1	*Zea mays*	ZEAMMB73_827243 (AFW63577.1)	Genbank
Zea 2		ZEAMMB73_204423 (DAA57458.1)	
Oryza 1	*Oryza sativa* (Japonica)	Os02g0709900 (NP_001047893.1)	Genbank
Oryza 2		Os01g0767000 (NP_001044359.1)	
Sorghum 1	*Sorghum bicolor*	Sb04g030240.1	JGI
Sorghum 2		Sb03g035670.2	

* JGI: www.phytozome.net
GenBank: www.ncbi.nlm.nih.gov/genbank/
TAIR: www.arabidopsis.org
Ensembl Genomes: plants.ensembl.org/Arabidopsis_lyrata/
