# Supplementary material for: Arabidopsis CROWDED NUCLEI (CRWN) proteins are required for nuclear size control and heterochromatin organization
Source: BMC Plant Biol. 2013 Dec 5;13:200. doi: 10.1186/1471-2229-13-200 (PMC3922879; doi:10.1186/1471-2229-13-200)
Supplement: Additional file 2 — Amino acid sequences comprising the extreme C-termini of 28 CRWN-like proteins, including ten CRWN4-like proteins. The similarity in this region, which falls outside of the coiled-coil domains, reinforces the topology of the tree shown in Figure 1. All of the proteins within the CRWN1-like clade, as well as the Physcomitrella homologs, contain a conserved C-terminal motif and a group of acidic residues approximately 25 amino acids from the end of the protein. Monocot CRWN4-like proteins contain a region with similar features but these conserved motifs are absent in CRWN4-like proteins from dicots (denoted by the yellow oval in Figure 1). [file 1471-2229-13-200-S2.pdf]

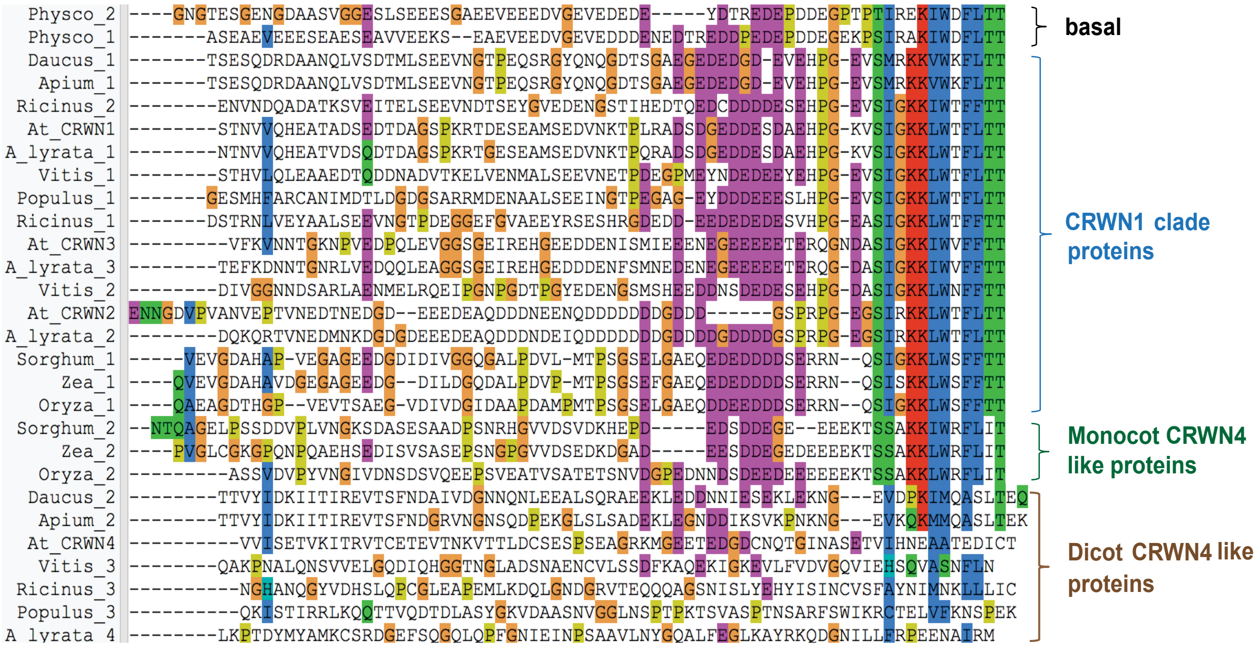

Physco_2 ----GNGTESGENGDAASVGGESLSEEESGAEEVEEEDVGEVEDEDE-----YDTREDEPDDEGPTPTIREKIWDFLTT
Physco_1 -------ASEAEVEEESEAESEAVVEEKS--EAEVEEDVGEVEDDDENEDTREDDPEDEPDDEGEKPSIRAKIWDFLTT
Daucus_1 -------TSESQDRDAANQLVSDTMLSEEVNGTPEQSRGYQNQGDTSGAEGEDEDGD-EVEHPG-EVSMRKKVWKFLTT
Apium_1 -------TSESQDRDAANQLVSDTMLSEEVNGTPEQSRGYQNQGDTSGAEGEDEDGD-EVEHPG-EVSMRKKVWKFLTT
Ricinus_2 --------ENVNDQADATKSVEITELSEEVNDTSEYGVEDENGSTIHEDTQEDCDDDDESEHPG-EVSIGKKIWTFFTT
At_CRWN1 --------STNVVQHEATADSEDTDAGSPKRTDESEAMSEDVNKTPLRADSDGEDDESDAEHPG-KVSIGKKLWTFLTT
A_lyrata_1 --------NTNVVQHEATVDSQDTDAGSPKRTGESEAMSEDVNKTPQRADSDGEDDESDAEHPG-KVSIGKKLWTFLTT
Vitis_1 --------STHVLQLEAAEDTQDDNADVTKELVENMALSEEVNETPDEGPMEYNDEDEEYEHPG-EVSIGKKLWTFLTT
Populus_1 -------GESMHFARCANIMDTLDGDGSARRMDENAALSEEINGTPEGAG-EYDDDEEESLHPG-EVSIGKKLWTFLTT
Ricinus_1 -------DSTRNLVEYAALSEEVNGTPDEGGEFGVAEEYRSESHRGDEDD-EEDEDEDESVHPG-EASIGKKLWTFFTT
At_CRWN3 ---------VFKVNNTGKNPVEDPQLEVGGSGEIREHGEEDDENISMIEEENEGEEEEETERQGNDASIGKKIWVFFTT
A_lyrata_3 --------TEFKDNNTGNRLVEDQQLEAGGSGEIREHGEDDDENFSMNEDENEGEEEEETERQG-DASIGKKIWVFFTT
Vitis_2 --------DIVGGNNDSARLAENMELRQEIPGNPGDTPGYEDENGSMSHEEDDNSDEDESEHPG-DASIGKKLWNFFTT
At_CRWN2 ENNGDVPVANVEPTVNEDTNEDGD--EEEDEAQDDDNEENQDDDDDDDGDDD------GSPRPG-EGSIRKKLWTFLTT
A_lyrata_2 --------DQKQRTVNEDMNKDGDGDEEEDEAQDDDNDEIQDDDDDDDGDDDDGDDDDGSPRPG-EGSIRKKLWTFLTT
Sorghum_1 -----VEVGDAHAP-VEGAGEEDGDIDIVGGQGALPDVL-MTPSGSELGAEQEDEDDDDSERRN--QSIGKKLWSFFTT
Zea_1 ----QVEVGDAHAVDGEGAGEEDG--DILDGQDALPDVP-MTPSGSEFGAEQEDEDDDDSERRN--QSISKKLWSFFTT
Oryza_1 ----QAEAGDTHGP--VEVTSAEG-VDIVDGIDAAPDAMPMTPSGSELGAEQDDEEDDDSERRN--QSIGKKLWSFFTT
Sorghum_2 --NTQAGELPSSDDVPLVNGKSDASESAADPSNRHGVVDSVDKHEPD-----EDSDDEGE--EEEKTSSAKKIWRFLIT
Zea_2 ----PVGLCGKGPQNPQAEHSEDISVSASEPSNGPGVVDSEDKDGAD-----EESDDEGEDEEEEKTSSAKKLWRFLIT
Oryza_2 ---------ASSVDVPYVNGIVDNSDSVQEEPSVEATVSATETSNVDGPEDNNDSDEEDEEEEEEKTSSAKKLWRFLIT
Daucus_2 --------TTVYIDKIITIREVTSFNDAIVDGNNQNLEEALSQRAEEKLEDDNNIESEKLEKNG---EVDPKIMQASLTEQ
Apium_2 --------TTVYIDKIITIREVTSFNDGRVNGNSQDPEKGLSLSADEKLEGNDDIKSVKPNKNG---EVKQKMMQASLTEK
At_CRWN4 ----------VVISETVKITRVTCETEVTNKVTTLDCSESPSEAGRKMGEETEDGDCNQTGINASETVIHNEAATEDICT
Vitis_3 --------QAKPNALQNSVVELGQDIQHGGTNGLADSNAENCVLSSDFKAQEKIGKEVLFVDVGQVIEHSQVASNFLN
Ricinus_3 ----------NGHANQGYVDHSLQPCGLEAPEMLKDQLGNDGRVTEQQQAGSNISLYEHYISINCVSFAYNIMNKLLLIC
Populus_3 ----------QKISTIRRLKQQTTVQDTDLASYGKVDAASNVGGLNSPTPKTSVASPTNSARFSWIKRCTELVFKNSPEK
A_lyrata_4 --------LKPTDYMYAMKCSRDGEFSQGQLQPFGNIEINPSAAVLNYGQALFEGLKAYRKQDGNILLFRPEENAIRM
basal
CRWN1 clade proteins
Monocot CRWN4 like proteins
Dicot CRWN4 like proteins
