# Supplementary material for: Arabidopsis CROWDED NUCLEI (CRWN) proteins are required for nuclear size control and heterochromatin organization
Source: BMC Plant Biol. 2013 Dec 5;13:200. doi: 10.1186/1471-2229-13-200 (PMC3922879; doi:10.1186/1471-2229-13-200)
Supplement: Additional file 3 — Transcript analysis of the crwn3-1 and crwn4-1 alleles used in this study. Reverse transcription-PCR results investigating the effect of T-DNA insertions on the transcription of CRWN3 and CRWN4. Panel A shows that a CRWN3 transcript is produced from the wild-type allele but not from the crwn3-1 allele using primers spanning the T-DNA insertion site. Panel B demonstrates that some transcription can be detected downstream of the insertion site from the crwn3-1 allele using RT-PCR and a primer set recognizing sequences 3’ of the insertion site. Panel C indicates that the T-DNA insertion in the crwn4-1 allele blocks transcription. Amplification of cDNA from cyclophilin and Actin2 were used as positive controls. M, marker lanes; + RT (plus reverse transcriptase); - RT (no reverse transcriptase). Information on the oligonucleotide primers used in these experiments is shown at the bottom of the figure. Our previous data [5] indicated that the crwn1-1 and crwn2-1 alleles block transcription downstream of the T-DNA insertion site in the sixth exon of both genes. [file 1471-2229-13-200-S3.pdf]

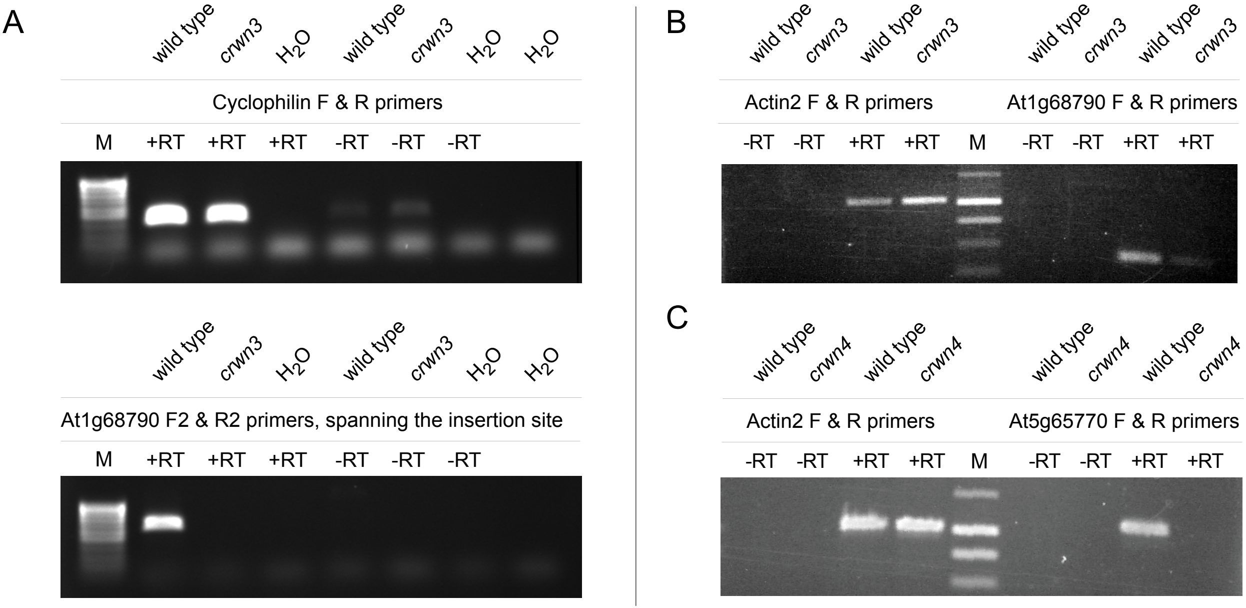

Gene/GI	Allele/T-DNA	Insertion Site	RT-PCR Primers	Position of primers
CRWN3<br>At1g68790	*crwn3-1*<br>SALK_099283	6th exon	F 5'-AGTGAACAGGCAGCTGGTGATAGT-3'<br>R 5'-ACTTCCAACTGCGGATCTTCGACT-3'<br>F2 5'–TCTCCTTCACGGTTTTGAGC–3'<br>R2 5'–GAGAAGCACATGAGGCAGTGT–3'	the 6th exon<br>the 8th exon<br>the 6th exon<br>the 4th exon
CRWN4<br>At5g65770	*crwn4-1*<br>SALK_079296	6th exon	F 5'-TCGCTAAACCGAGAGCGTGAAGAA-3'<br>R 5'-TTGGTCACCTCTGTCTCACACGTT-3'	the 6th exon<br>the 7th exon
Actin 2			F 5'-TGATATTCAACCAATCGTGTGTGAC-3'<br>R 5'-AAGCAAGAATGGAACCACCGATCC-3'	the 1st exon<br>the 2nd exon
Cyclophilin			F 5'–CGATAAGACTCCCAGGACTGCCGA–3'<br>R 5'–TCGGCTTTCCAGATGATGATCCAACC–3'	the only exon<br>the only exon
