# Supplementary material for: Arabidopsis CROWDED NUCLEI (CRWN) proteins are required for nuclear size control and heterochromatin organization
Source: BMC Plant Biol. 2013 Dec 5;13:200. doi: 10.1186/1471-2229-13-200 (PMC3922879; doi:10.1186/1471-2229-13-200)
Supplement: Additional file 4: Table S2 — The nuclear phenotype data for crwn mutants used to construct Figure 4 is displayed in tabular form. The average endopolyploid level (ave. ploidy level) was determined by flow cytometry as described in Methods. The actual measurements were converted to relative measurements (fraction of wt, third column) using the wild type (wt) values for normalization. The average nuclear size (ave. nuclear size ± standard error of the mean) corresponds to data from Figure 4A. The fifth column normalizes these size values to the wild type values. [file 1471-2229-13-200-S4.pdf]

	ave. ploidy level	fraction of wt	ave. nuclear size (μm^2)	fraction of wt
wild type	9.74	1.00	73.0 ± 1.18	1.00
crwn1	9.52	0.98	35.7 ± 1.40	0.49
crwn2	8.42	0.86	72.1 ± 2.71	0.99
crwn3	8.41	0.86	69.8 ± 3.43	0.96
crwn4	9.52	0.98	39.3 ± 2.20	0.54
crwn1 crwn2	6.91	0.71	11.8 ± 0.78	0.16
crwn1 crwn3	7.92	0.81	26.5 ± 1.14	0.36
crwn1 crwn4	8.40	0.86	27.8 ± 1.09	0.38
crwn2 crwn3	8.85	0.91	72.9 ± 3.60	1.00
crwn2 crwn4	7.43	0.76	42.5 ± 1.43	0.58
crwn3 crwn4	8.66	0.89	38.1 ± 1.24	0.52
crwn1 crwn2 crwn4	6.31	0.65	10.3 ± 0.31	0.14
crwn1 crwn3 crwn4	5.16	0.59	11.2 ± 0.51	0.15
