# Supplementary figures and images for: Arabidopsis CROWDED NUCLEI (CRWN) proteins are required for nuclear size control and heterochromatin organization
Source: BMC Plant Biol. 2013 Dec 5;13:200. doi: 10.1186/1471-2229-13-200 (PMC3922879; doi:10.1186/1471-2229-13-200)

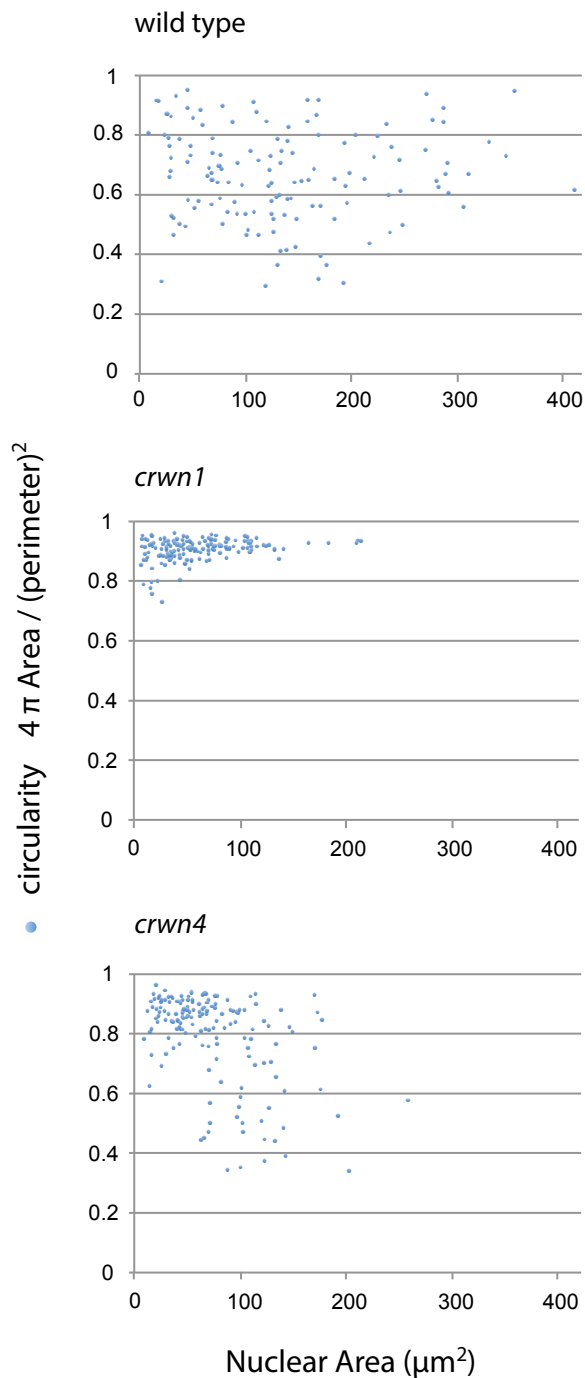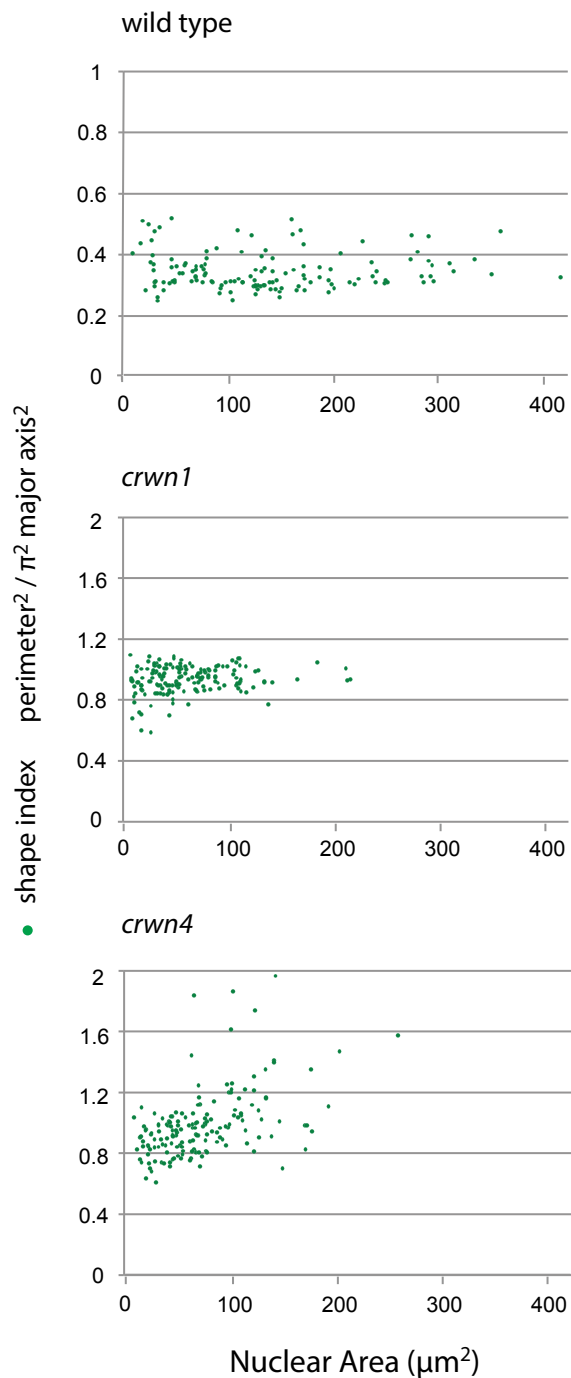

Supplement: Additional file 5 — Nuclear shape changes in crwn1 and crwn4 mutants. Images of representative DAPI-stained adult leaf cell nuclei from wild type, crwn1 and crwn4 mutants were processed by ImageJ software to determine the circularity index (4π · Area/(perimeter)2), as well as a shape index (perimeter/π · major axis)2. Nuclei that deviate from a perfect circle (1.0) show a lower circularity index. The shape index highlights different types of deviations from the round shape. Nuclei in the crwn1 sample show a shape index close to 1.0, indicating consistently round nuclei. The reduced (relative to 1.0) shape indices in the wild-type sample across all nuclear sizes indicate uniformly elongated nuclear shapes. The elevated shape indices characteristic of larger crwn4 nuclei result from the presence of thin projections from the surface of otherwise round nuclei. [file 1471-2229-13-200-S5.pdf]

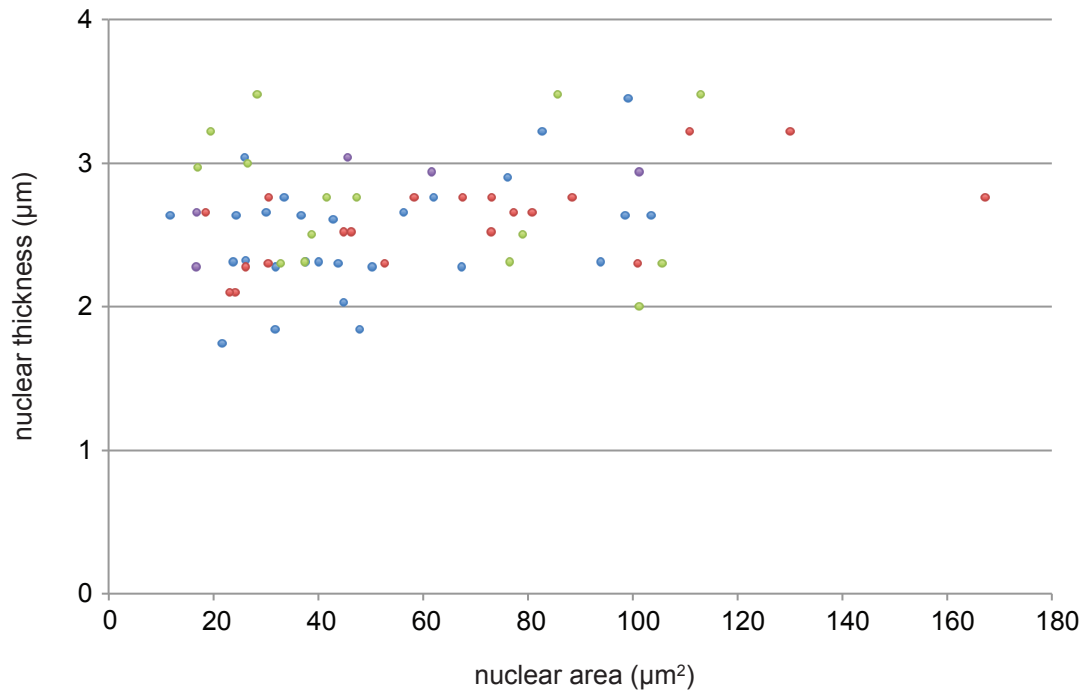

Supplement: Additional file 6 — Leaf nuclear preparation and confocal imaging reveals a consistent nuclear thickness across a range of nuclear sizes. Mature leaves were harvested from five individuals in a F2 population segregating both crwn1 and crwn2 mutations (F2 plants of a crwn1 crwn2 x wild type cross). Consequently, the sample captured a range of nuclear shapes and sizes. The nuclei were fixed, isolated, and prepared for imaging as described for Figure 4. Following DAPI staining, the three-dimensional signal of different nuclei were recorded and reconstructed using a Leica SP5 confocal microscope. The area of each nucleus was measured using ImageJ, while the thickness of each nucleus was determined by the number and thickness of steps on the z-axis necessary to move from the top to the bottom of each nucleus. The different colored dots on the graph correspond to different slides imaged in this experiment. The results indicate that our preparation and imaging procedure generates nuclei with a relatively uniform thickness, mostly in the 2–3 micrometer range, regardless of the size and shape of the nuclei. Further, this thickness is consistent across individual slides. [file 1471-2229-13-200-S6.pdf]

Chromocenter number in leaf cell nuclei

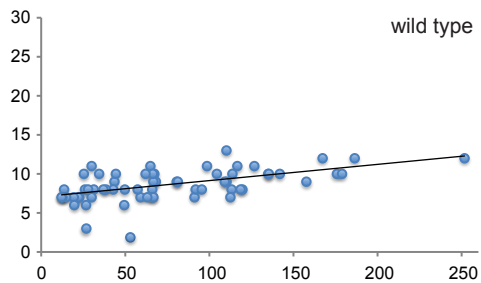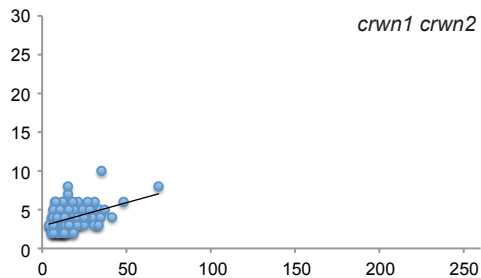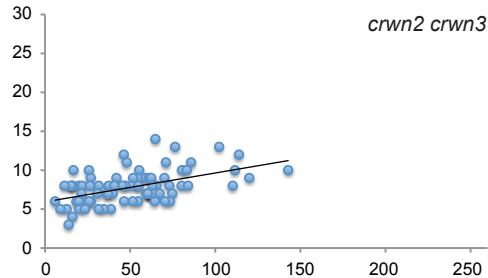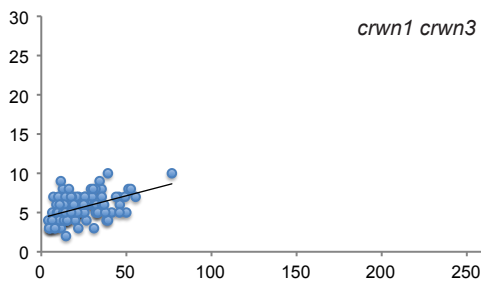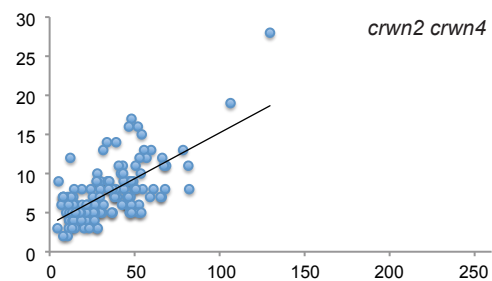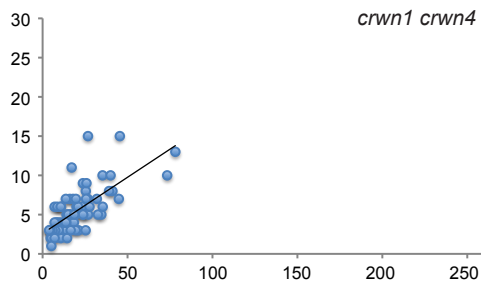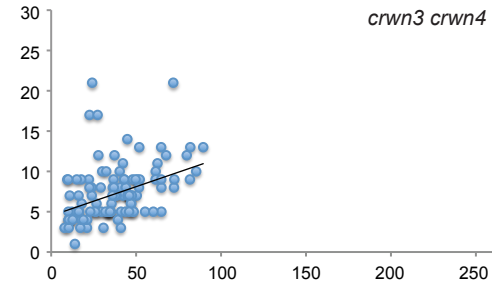

Nuclear area ( $\mu\text{m}^2$ )

Supplement: Additional file 7 — Chromocenter changes in crwn double mutants. Nuclei were harvested from developmentally matched rosette leaves from approximately one month-old plants, stained with DAPI, and imaged using epifluorescence microscopy. The area and chromocenter number of randomly-selected individual nuclei (n = 41–52) were determined for each genotype, and chromocenter number was plotted against nuclear area. A linear regression line showing the relationship between chromocenter number and nuclear size (as a proxy for endopolyploidy level) was plotted for each genotype. [file 1471-2229-13-200-S7.pdf]
